# Environment Context Variability and Incidental Word Learning: A Virtual Reality Study

**DOI:** 10.3390/brainsci12111516

**Published:** 2022-11-08

**Authors:** Francisco Rocabado, Jorge González Alonso, Jon Andoni Duñabeitia

**Affiliations:** 1 Centro de Investigación Nebrija en Cognición (CINC), Universidad Nebrija, 28248 Madrid, Spain; 2 AcqVA Aurora Center, UiT The Arctic University of Norway, 9019 Tromsø, Norway

**Keywords:** incidental learning, virtual reality, context variability, context-dependent memory

## Abstract

Previous research has shown that changes in the scenarios in which something is learned and recalled, respectively, may result in a subpar performance in memory recollection. The current study aimed to evaluate how changes in the visuo-perceptual environmental learning context impact incidental vocabulary learning. To this end, a highly immersive virtual reality setting was created, and participants were required to read eight distinct stories visually presented to them. A novel word was delivered twice in every paragraph and embedded in each story. Stories could be displayed either in a high variability condition, where each paragraph was shown in a new environment context (four different classrooms) or in a low variability condition, where each paragraph was shown in the same context. The findings obtained across four assessment tasks (free recall, recognition, picture matching, and sentence completion) demonstrated that significant visuo-perceptual variability did not bring about any disadvantages in word learning. Thus, perceptual information from a physically diverse environment could provide a variety of instructional and educational beneficial possibilities in the absence of a learning disadvantage.

## 1. Introduction

The strategy of retracing one’s steps to retrieve a lost thought relies on our ability to recreate the same environmental conditions in which a memory was encoded. Consider being at your office desk, working on your next article, and deciding to go and get a chocolate treat from the vending machine on the ground floor. When you get to the vending machine, you realize you have completely forgotten why you went there in the first place. However, if you go back to your desk, you will probably remember what it was you wanted from the vending machine. This anecdotal observation by which (re)experiencing a familiar context after some time can bring back a significant number of forgotten memories is known as the environmental reinstatement effect [1,2]. This effect is mostly present in the context-dependent memory literature, which refers to the increased recall of specific episodes or information when the context is the same at encoding and retrieval. Although Jansen et al. [3] provided the first evidence showing a context-dependent influence on memory for nonsense syllables, the first notable study to report a strong effect of context in learning and recall was the well-known scuba diver experiment [4]. 

*Context* has been described and operationalized in a variety of ways within the context-dependent memory literature, for instance, as changes in maze orientation and lighting that interferes with animals’ performance [5]; posture during learning [6]; in the form of audience vs. no audience [7], and even as a state of mind connected to the presence or absence of drugs [8]. However, in Godden and Baddeley’s study [4], participants were asked to learn words either on land or underwater and were then tested in one of the two environments, showing better word recall when the environmental context of recall matched the learning context. While impressive, these results have not often been successfully replicated (see [9] for an early review). Some authors have suggested that the type of contextual modifications used in these replications (e.g., screen background color, physical rooms) are potentially too small to generate contextual episodic memory traces, therefore failing to reproduce the type of stark contrast of the original scuba diver study, where completely different bodies of knowledge or schemas were likely activated in each environment [10]. 

While the studies mentioned above refer to context variations between the learning and testing environments, a different line of research has focused on how variability within the learning context could impact learning. In this line, several recent studies on incidental vocabulary learning have highlighted that contextual diversity, understood as the number of different unique contexts in which a word is encountered, aids vocabulary acquisition (e.g., [11])—note that context typically refers to the kind of document a word is embedded in [12,13]. Based on the principle of likely need, Adelman et al. [12] established the theoretical support for the importance of contextual diversity in lexical organization (see [14] for a thorough discussion). This principle is founded on the idea that human memory is adaptable and should thus be arranged to maximize access to relevant information [15]. In this sense, a word that has been encountered in a variety of circumstances during learning is more likely to be required in an unknown future scenario, thus becoming more readily available in the lexicon. 

This mirrors the development of object categorization, which depends on recognizing a single object across different visual backgrounds (e.g., [16,17]). 

In studying the role of contextual diversity in vocabulary acquisition, Tapia et al. [18,19] found that children learned more words incidentally when these were encountered in a high diversity context (e.g., texts of natural science, math, fables) compared to a low diversity context (e.g., only texts of natural science). Furthermore, Frances et al. [20] found that higher contextual diversity improves the performance of incidental vocabulary learning in adults and that this benefit can be translated to foreign language learning scenarios. Furthermore, in the last two decades, the value of contextual diversity as a relevant variable in other domains has been empirically supported. It has been shown that contextual diversity facilitates the learning of new information [21], facilitates the acquisition and processing of new vocabulary in childhood [22,23], and enhances foreign language vocabulary learning [20]. Moreover, contextual diversity has been shown to have a strong effect on word identification latencies in monolinguals [12,24] and second language speakers [25], as well as reading times [26]. On par, a specific event-related potential signature on word recognition has also been reported in contrast to the evoked potential produced when word frequency is accounted for [27], thus highlighting the relevance and specificity of contextual diversity as a cue to word processing and learning.

This benefit of contextual diversity on new vocabulary learning in both children and adults has been explained in terms of semantic diversity, based on proposals such as Jones et al.’s [28], and the notion of contextual diversity was adapted to also incorporate the effects of the variability of the semantic content and discourse context [13,28,29]. One of the main premises of Jones et al.’s [28] model is that if context repetition matters, these effects should be circumscribed to the number of various context types in which a word appears rather than to the overall number of instances (namely, a type-token distinction). When one learns the kinds of circumstances in which a word is likely to appear, that word may be anticipated when one enters those contexts—meaning that the word becomes more available and ready to be used. However, if a word occurs in an indiscriminate manner across distinct context types, it cannot be anticipated and should thus be strongly retained in memory and its mental representation updated. Thus, after our first encounter with a word, subsequent encounters over different contexts will result in a richer lexical portrayal. 

This is similar to the Semantic Distinctiveness Model or Bolger et al.’s [30] Contextual Variability Hypothesis. Similarly, Bolger et al. [30] argue that concept learning is enhanced when concepts appear in variable as opposed to uniform contexts. When a word is experienced, different memory traces are created, including episodic information of the context it has been encountered in. Different contexts entail different episodic experiences, which increases the number of memory traces through which the word can be retrieved [31].

As we see, theoretical proposals have highlighted the role of semantics in vocabulary acquisition, as well as the enhancement in word learning and recall provided by episodic memory traces. This raises the reasonable question of how context-dependent memory effects (i.e., the context reinstatement effect) interact with this type of learning condition, namely, a manipulation of contextual diversity since context-dependent memory effects also rely on episodic information during encoding. 

A recent study by Shin et al. [32] carried out a replication of the context reinstatement effect in virtual reality (VR) environments. VR settings allow for enough variation to reproduce the vast contrast between the on-land and underwater environments in Godden and Baddeley’s study [4], thus overcoming the limitations of the relative uniformity of a laboratory setting that previous research had encountered. In their case, the chosen environments were underwater and on the surface of Mars. Two findings by Shin et al. [32] are particularly worth noting. The first is that they replicated the context reinstatement effect, showing that words were better recalled when the environments of learning and recall were matched. The second is that effect size was not constant across items but rather varied as a function of how context-relevant those items were judged to be when encountered during the learning phase (rated in terms of “useful” vs. “harmful” in the VR game). This last finding provides further support to the idea that the larger bodies of knowledge or schemas associated with a given learning context are relevant for the context-dependent memory effect and that words/items deemed to “belong” to those schemas are more easily integrated into them and thus recalled. This highlights the tight relationship between memorization and its context during the learning process, whereby the physical context also provides important episodic cues in the learning of new content.

However, other characteristics of the learning environment have been shown to have an impact on modulating the creation of new memory traces. In this sense, Tapia et al. [33] showed that semantics is not the only aspect of a word’s context involved in the facilitative effect of contextual diversity. In their experiment, diversity was manipulated as variability in the perceptual characteristics of voices from different narrators. Children listened to narrations recorded by a single speaker (low-diversity condition) or multiple speakers (high-diversity condition). The novel words embedded in these recordings were significantly better learned when they were presented by multiple narrators compared to recordings with a single narrator. Importantly, the semantic context in which words appeared was held constant across the low- and high-diversity conditions, so the effect of contextual diversity in the study cannot be ascribed to semantic variability. The idea that episodic memory traces associated with the perceptual characteristics of the learning environment are part of the enhancement provided contextual diversity links back to Bolger et al. [30] and provided further support for theories of lexical access and representation that assume a critical role of the encoding of episodic memories (e.g., [34]).

Yet, in light of the findings of Shin et al. [32], it is reasonable to ask whether variation in the perceptual characteristics of a prototypical learning context (i.e., a classroom) may have negative repercussions. In fact, altering learning spaces between school subjects is a very rare practice in school, where most lessons are taught in the same classroom—with some notable exceptions that might require specific facilities such as Music, Sports, or Technology. In the present study, we sought to investigate to what extent pseudorandom perceptual variation in the physical learning environment modulates incidental word learning. Based on the literature provided on vocabulary acquisition in school settings [18,19,33] and proficient readers [20], we expect to see a similar learning advantage aided by diverse context manipulation. To achieve this, we used virtual reality (VR) technology and exposed participants to text passages containing target pseudowords to be learned in two degrees of contextual variability scenarios: high (across four different physical environments) or low (within a single physical environment). Word learning was then evaluated outside of the VR environment through four different recall and recognition tasks.

## 2. Materials and Methods

### 2.1. Participants

Forty-six undergraduate and graduate students from Nebrija University in Madrid, Spain, participated in the study. These were 31 females with ages ranging between 18 and 52 years old (Mage = 25.90, SD = 8.08) and 15 males with an 18–31 age range (Mage = 23.86, SD = 4.10). All participants had normal or corrected-to-normal vision, no signs of cognitive dysfunction as measured by the Cognitive Assessment Battery (CAB)™ PRO (CogniFit Inc., San Francisco, CA, US), and were naive to the purpose of the experiment. Prior to the beginning of the experimental session, participants were prompted to read and sign an informed-consent form for data collection.

### 2.2. Materials

The experimental material consisted of the same eight pseudowords employed in the experiment by Frances et al. (2020) on contextual diversity and incidental word learning. However, opposite to Frances et al., these were embedded across eight different stories. Each pseudoword was placed in substitution of a high-frequency word that was the most representative of its semantic category. This allowed participants to quickly infer its meaning within the narrative by matching it to the known meaning of the substituted real word (e.g., OTILA was used as a substitution for the word APPLE, and ESIBA was used to replace HOUSE). A further advantage of the word substitution method is that associating the meaning of the pseudoword to a real word allows the former to take on visual depictions of the latter as well, which creates opportunities for testing learning beyond simple word recall—e.g., through a picture-word matching task. For instance, OTILA would be described as something that can be eaten, that contains seeds in its core, that may also host worms, that is healthy, and that, as in the case of oranges, one could make juice out of it. Therefore, participants would be able to create a representation of something apple-like, if not an apple (see https://doi.org/10.6084/m9.figshare.21485619 (accessed on 4 November 2022) to access all Supplementary Materials). 

Although using real words to describe our pseudowords might arouse the salience of the latter, participants were not explicitly asked to learn the pseudowords (hence target words) during the training phase, and no mention was made of the subsequent testing tasks in order to strengthen the incidental nature of participants’ learning process. Instead, participants were given instructions to read each of the texts at their own pace. To ensure that participants paid attention while reading, they were informed that after every story, a yes/no comprehension question would be asked. By doing this, we hid the aim of the task by making participants focus on comprehending each text whilst ensuring incidental exposure to the pseudowords. This allowed us to avoid any explicit learning goal [30] (see [20,30,35] for incidental learning approaches of pseudowords). 

Each target word was embedded within eight different narrative stories (one word per story). Every text was the result of four knitted paragraphs of thirty-five letters of length each. The target word was embedded two times within every paragraph. Thus, participants were exposed to each target word eight times. All potentially informative clues regarding our target words were controlled across all embedding texts.

Contextual diversity in the learning contexts was manipulated within participants, with half of the stories presented in a high-variability context (i.e., across four physically different environments) and the other half in a low-variability context (i.e., a single physical environment). Similar to Tapia et al. [33], we maintained the number of possible contexts participants were exposed to constant. In our design, each of the target words on the low variability condition was matched with one of the four available classroom models. All classroom context presentations on the high-variability condition and classroom pairings for the low-variability condition were randomized. Finally, two lists were created so that each story could appear in the two variability conditions across participants in a counterbalanced manner. Participants were randomly assigned to one of the two experimental lists.

### 2.3. Environments

The training and evaluation phases took place in the same experimental session, separated by an unrelated distractor task (i.e., math exercises). The training phase took place within a virtual reality setting through an HTC VIVE Pro head-mounted display (HMD). The evaluation phase was undertaken in a traditional laboratory setting on a 2D screen monitor.

**Figure 1:**
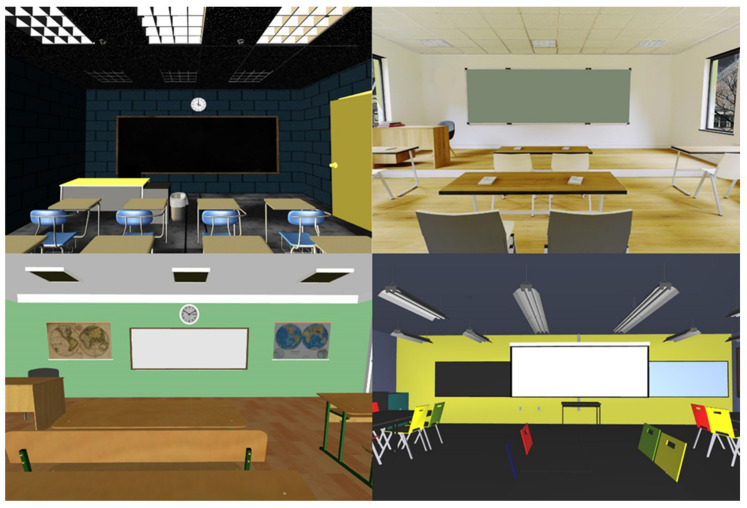
Four physical VR environments.

Four different open-access 3D Classroom models found at Turbosquid and Sketchfab were downloaded for use in the experiment. Models that included furniture such as student desks or blackboards and were available in an editable format were deemed suitable for downloading. Each model was completely different from the others in terms of size, shape, color, decoration, and furniture distribution. In order to improve participants’ immersion in the virtual reality environment, HDRi backgrounds were included in some of the 3D models. Furthermore, unnecessary 3D child objects within the parent model were removed with Blender [36]. Figure 1 shows the four different environments used in the experiment.

### 2.4. Apparatus

The virtual reality setting was programmed in Vizard 6 [37], a Python-based software (version 2.7; Python Software Foundation, https://www.python.org/, (accessed on 4 November 2022)). The experiment script was executed on a high-end gaming laptop computer equipped with an Intel Core i7-10750H (2.6 Hz), running the Windows 10 operating system (64 bit), 32 GB RAM, and an NVIDIA GeForce RTX 2070 video card. To ensure high-performance device communication between the computer and the HMD, battery-saving settings were always disabled.

Three-dimensional stimuli classroom models and experimental material were presented through the HTC VIVE Pro HMD [38] at 2880 × 1600 pixel resolution (1440 × 1600 per eye) and a 90-Hz refresh rate. Eye-tracking data was not collected nor recorded during the training session; however, to ensure participants’ device suitability, the automatic eye-tracking calibration interface was employed so the headset was well-positioned on the face, and the eye-to-eye distance was adjusted on every participant. Furthermore, the Motion Smoothing system in SteamVR for HTC Vive Pro was disabled to ensure a constant refresh rate frame during the tasks. 

Finally, in order to signal changes between paragraphs and scenes, an all-white neutral space was presented. Participants’ camera position within the VR environment was fixed to its axis, allowing us to maintain a constant distance to the blackboard yet allowing participants to explore their surroundings by rotating on themselves.

### 2.5. Task and Procedure

The training phase and the test phase were carried out individually. A distractor task was implemented between phases. Participants were randomly assigned to one of the experimental set conditions. Before starting the training, participants were informed and instructed about the calibration interface. Moreover, participants were given two controllers, which during the VR immersion, would turn into visible virtual hands within the VR environment. Once the headset was placed, participants had to make sure that each virtual hand was held with the corresponding hand.

During the VR immersion, participants were first given 15 s to explore their surroundings. After this time, an auditory signal would mark the beginning of the reading time, and a text paragraph would be displayed. Participants had to read all the stories presented to them, one paragraph at a time, at their own pace. To move forward between paragraphs, participants had to pull one of the triggers in each controller. After four paragraphs, a comprehension question was displayed. To provide an answer, participants had to pull one of the corresponding triggers: right for yes, and left for no.

Every paragraph was presented in written form on a board. Text distribution, size, and position were constant in both learning conditions (high and low variability). To signal and delimit the transition between paragraphs, an all-white neutral space was presented for 2500 ms. Once a story was read and its comprehension question response was provided, the beginning of a new story was marked by a three-second countdown accompanied by a beep sound every second.

Once all stories were read, the VR headset was removed, and participants had to complete an unrelated distractor task, which was composed of different math exercises. Participants were instructed to answer quickly as many exercises as they could in five minutes, trying to be as accurate as possible. Exercises consisted of mathematical additions and subtractions. When participants finished the distractor task, they were informed for the first time that they had to complete a series of additional tasks to measure incidental word learning. This phase consisted of a free recall task, an old-new recognition task, a picture-word matching task, and a sentence completion task (similar to those used in [20,33]).

For the free recall task, participants were asked to recall and write all the new words they remembered encountering while reading the texts. In the old-new recognition task, 24-word-like strings were displayed on a computer. Participants were instructed to mark each of the strings as “seen” or “not seen,” depending on whether they believed these had appeared during the reading phase. These twenty-four stimuli were divided into three categories: old when the presented stimuli matched the ones presented during the reading phase (i.e., OTILA), new-similar when the presented stimuli were the result of replacing the middle or last consonant of an old stimulus (i.e., OTIBA instead of OTILA), and new when the presented stimuli belonged to a new and unrelated set of pseudowords (i.e., REJUCA instead of OTILA). 

After the old-new recognition task, participants completed a picture-word matching task. For this task, picture materials were required to pair our target stimuli with real object drawings. These were chosen from the MultiPic database [39,40], depicting the meaning behind the experimental stimuli presented in the texts—with the exception of “water,” which was replaced with the pictogram for a water tap. An extra set of eight more pictograms that depicted a semantic competitor were also selected from the same database to create a “competitor” category (i.e., the picture of an orange instead of an apple). In this task, both pictures and words were centered vertically and horizontally on the screen, with the pictures above the target pseudoword stimuli. With these materials, a total of 32 picture and word pairings were created in four conditions (i.e., eight trials per condition): match, when the picture represented the meaning of the target pseudoword (i.e., the picture of an apple paired with OTILA); competitor, when the picture was semantically related to but did not depict the target pseudoword (i.e., the picture of an orange paired with OTILA); distractor, when the picture depicted the meaning of a pseudoword different to the one it was paired with (i.e., the picture of a house (ESIBA) paired with OTILA); and unrelated, when the picture depicted the meaning of a semantically unrelated word (i.e., the picture of a roof paired with OTILA). To create the unrelated condition, each pseudoword was paired with the competitor picture of one of the pseudowords seen during the reading phase (e.g., the picture roof would be the competitor of the pseudoword ESIBA, which would match with a house depiction). This allowed us to generate the right conditions without increasing the overall semantic space of the whole task. In each trial, participants were asked to indicate whether the picture-pseudoword pairing was correct.

Finally, a word completion/cloze task was presented. Here, participants would encounter a total of 32 sentences that were present in the stories (e.g., “juice can be made from OTILA”). A total of four sentences per target word were used. Every sentence contained a blank space to be filled with one out of four provided pseudoword options. Two were part of the experimental material (e.g., OTILA and ESIBA)—with only one being correct, and the other two pseudowords were the result of a letter replacement from within the other two options (e.g., OTIBA and ESILA).

Stimuli presentation in all tasks was randomized. Both the intermediate distractor task and the evaluation phase took place in a traditional 2D laboratory setting, on a desktop computer through Gorilla Experiment Builder [41] and executed within the same web-based platform.

## 3. Results

Collected data were cleaned using R [42] in the RStudio integrated environment [43]. Descriptive statistics of accuracy scores across tasks and experimental manipulations are reported in Table 1. Timed-out trials were removed from the accuracy analysis. Statistical analyses were conducted in JASP [44]. The factor List was included as a dummy between-participants variable in the ANOVAs to extract the variance due to the error associated with the lists.

### 3.1. Comprehension Check and Reading Times during Learning

Participants’ accuracy of comprehension questions was high (M = 0.94 SD = 0.237). Statistical differences between context variability conditions were assessed. Since the normality test was significant (*p* > 0.001), a Wilcoxon’s signed-rank test showed no statistical difference between high contextual variability (Mdn = 1) compared to low contextual variability (Mdn = 1) scores, W = 40, *p* = 0.239.

The participants’ average reading time per paragraph was 13.67 s (SD = 6.94). The comparisons showed no statistical differences between the reading times in the high contextual variability (M = 13.87 s, SD = 4.61) and the low contextual variability conditions (M = 13.47 s, SD = 3.83), t(45) = 1.22, *p* = 0.229, MDiff = 0.40 s).

### 3.2. Free Recall Task

First, differences in the mean recall between conditions were analyzed. Items in the high contextual variability condition were recalled with a relatively low mean accuracy (M = 0.141, SD = 0.240), and the same was true for the items in the low contextual variability condition (M = 0.125, SD = 0.202). No significant differences were observed between the two conditions (F [1, 44] = 0.396, *p =* 0.533, η2 = 0.004). Participants’ overall free recall scores were low, which is indicative that the incidental nature of the task remained, as participants’ scores show no learning benefit. Thus, participants were not impaired nor benefited by the presentations of items across a wide range of physical environments. Furthermore, we conducted Bayesian Null Hypothesis testing comparisons [45,46] to further explore the effect of contextual variability on recall. Bayesian paired sample t-tests of the null hypothesis (BF01), which provides an estimation of the evidence supporting the absence of differences across conditions, showed that the null hypothesis was able to explain the effects of accuracy scores 3.97 times better than the alternative hypothesis.

Additionally, this task was evaluated in a similar way as in Frances et al. [35]. Since approximate but not exact recalls are frequent in a free recall task, we relied on a derived standardized value from Levenshtein Distance that accounts for the similarity between a given answer and the expected string in terms of character insertions, deletions, and substitutions. By doing this, the degree of similarity of participants’ responses to the expected strings can be computed in 0-to-1 continuums, with 1 being a perfect match and 0 being a totally different pair of strings. Responses shorter than three letters and real words related to the target word were discarded, as they were not considered real attempts. Standardized accuracy scores showed no differences between high contextual variability (M = 0.201, SD = 0.108) and low contextual variability (M = 0.196, SD = 0.107) (F[1, 44] = 0.153, *p =* 0.698, η2 = 0.027). Furthermore, these low standardized scores are indicative that participants’ responses were highly different from the pseudowords they were expected to learn during the training phase. Similarly, the null hypothesis test showed that the null hypothesis (BF_01_) could explain the accuracy of data on contextual variability 4.36 times better than the alternative hypothesis.

### 3.3. Recognition Task

We carried out a three (Stimulus Type: old, new, and new-similar) by two (context variability: high vs. low) repeated-measures ANOVA. A significant main effect of stimulus type was found (F[2, 88] = 38.285, *p* < 0.001, η2 = 0.286), with responses to new stimuli being significantly more accurate than those to old stimuli (MDiff = −0.256 SE = 0.036, *p*_Holm_ < 0.001) and new-related stimuli (MDiff = −0.287 SE = 0.036, *p*_Holm_< 0.001). However, context variability did not yield a main effect (F[1, 44] = 0.296, *p* = 589, η2 = 0), nor did the interaction between the main factors (F[2, 88] = 1.181, *p* = 0.312, η2 = 0.004). These results suggested that participants’ performance across conditions was not modulated by the context variability manipulation. 

To assess the effect of context variability further averaged across our stimulus types, we conducted Bayesian Null Hypothesis testing [45,46]. Results of the Bayesian analysis showed that the null hypothesis (BF_01_) was able to explain the effects of accuracy scores 5.32 times better than the alternative hypothesis, reinforcing the view of an absence of differences across conditions.

### 3.4. Picture Matching

Three different comparison analyses were performed between conditions to assess participants’ response accuracy: match vs. competitor, match vs. distractor, and match vs. unrelated. Due to task coding issues, data associated with the pseudoword “tesid” had to be removed from the analyses.

A four (Stimulus Type: match, competitor, distractor, unrelated) by two (context variability: high vs. low) repeated measures ANOVA was carried out. A significant effect was found for Stimulus Type (F[3, 132] = 17.939, *p* < 0.001, η2 = 0.145). Post Hoc analyses showed that match stimuli were significantly better recognized than distractor stimuli (MDiff = 0.158, SE = 0.041, p_Holm_< 0.001) and unrelated stimuli (MDiff = −0.134, SE = 0.034, p_Holm_< 0.001). Suggesting that participants could reliably distinguish between the graphic representations of different target pseudowords among those presented during the learning phase and those that were never seen before, as expected. However, no significant differences were found between match stimuli and competitors (MDiff = −0.009, SE = 0.044, p_Holm_ = 0.838). This indicates that participants were unable to reliably distinguish between the matching representation and the semantic competitor, as illustrated by the incorrect association of both the picture of an orange (semantic competitor) and of an apple (match) to the pseudoword OTILA. Further comparisons showed that distractor stimuli were less likely to be interpreted as false in comparison to competitor stimuli (MDiff = −0.167, SE = 0.047, p_Holm_ = 0.002) and unrelated categories (MDiff = −0.292, SE = 0.042, p_Holm_ < 0.001). Finally, the competitor stimuli’s accuracy was significantly lower compared to that of unrelated stimuli (MDiff = −0.125, SE = 0.030, p_Holm_ < 0.001), as one would expect. Besides that, no significant effect was found for context variability (F[1, 44] = 0.032, *p* = 0.859, η2 < 0) nor an interaction (F[3, 132] = 1.193, *p* = 0.315, η2 = 0.011). 

The effect of context variability was further explored through Bayesian analysis by testing the null hypothesis, where variability was averaged across stimulus type. Results show that the null hypothesis (BF_01_) was able to explain the effect 6.96 times better than the alternative hypothesis.

### 3.5. Sentence Completion

Accuracy scores in the sentence completion/cloze task showed no differences between high contextual variability (M = 0.686, SD = 0.221) and low contextual variability contexts (M = 0.702, SD = 0.200), F[1, 44] = 0.351, *p* < 0.556, η2 = 0.002. 

The Bayesian Null Hypothesis testing showed that the null hypothesis (BF_01_) was able to explain the effects 3.91 times better than the alternative hypothesis.

## 4. Discussion

The present study investigated the role of perceptual variability, as manipulation of contextual variability beyond semantic distinctiveness, on the incidental learning of new vocabulary using different VR environments during the learning phase. Results from four different recall and recognition tasks showed that perceptual variability did not enhance (nor impair) incidental learning of the target pseudowords. This contrasts with results from previous research showing that contextual variability is a better predictor of word naming and lexical decision [12] as well as word learning in children [47]. Likewise, contextual variability has been shown to foster incidental vocabulary learning (e.g., [18,28,33]), both in a first and in a second language (e.g., [20]). While contextual diversity has most often been manipulated as semantic diversity, Tapia et al. [33] showed that perceptual diversity (variability in the narrator’s voice during critical passages) had similar effects on word learning and recall. The question, then, is why our manipulation of the perceptual environment of participants during the learning phase did not show the same or similar results.

A potential explanation lies in the ability of our manipulations to generate episodic memory traces that are sufficiently tied to the critical stimuli to enhance memorization and recall. As we mentioned in the introduction, episodic accounts of lexical representation and retrieval (e.g., [30,31,34]) assume that episodic memory traces associated with a speaker’s encounters with a given word become pathways to access that word and that a larger and more diverse amount of these traces improves the overall recoverability of that word. The literature on the context-dependent memory effect, which probes episodic memory accounts of lexical representation by investigating whether matching environments in learning and test phases improve recall, provides some cues on how efficient different degrees of perceptual variability may be in creating these episodic traces. Smith and Vela [9] argued that the usual laboratory manipulations (e.g., background color) might fail to sufficiently distinguish the perceptual environment of different stimuli, yielding null effects in context-dependent memory experiments. VR environments provide a powerful tool to increase the distinctiveness of our learning environments (e.g., [32]), but even within VR, one may argue that certain manipulations may be insufficient, explaining failures to replicate some of the findings (see Shin et al.’s [32] discussion of Wälti et al. [48]).

The notion of contextual diversity has been adapted to include new sources of variance, such as the variability of the semantic content and the discourse context [13,28,29]. Contextual diversity has been typically linked to a rational analysis of memory, which holds that memory is designed to store information that will be needed later (i.e., the principle of likely need). On this premise, physical variation can be included as a contextual source in language learning. However, one could argue that, based on the context-dependent learning and memory effects, variations of physical contexts in which a learning process takes place can hinder the creation of dependent-to-context learning, as this relies on the episodic cues also provided by the physical environment. From the literature on visual recognition development in children, we know that memory dependence on context is part of an evolutionary continuum that ends with memory independence through context variability [49]. In this sense, object-context dependency is much stronger during the first developmental stages, becoming less relevant as a direct function of development [49]. This developmental pattern is similar to that of language production, given that words evolve from context-dependent categories (e.g., the word *car* is only uttered when the object is seen under the same circumstances) to more abstract categories (e.g., the word *car* can be uttered when the object is seen on different contexts, or absent altogether) [50,51]. In this vein, one could hypothesize that context-dependent memory formation (and consequently contextual diversity at the physical environmental level) could aid word learning during the early stages of the developmental process, whereas this would not be the case in later developmental stages. In this sense, the negligible differences observed across contexts in the current study might well be a consequence of two opposing forces that neutralize each other. On the one hand, the literature on contextual diversity effects suggests that adding variability to the physical environment where learning takes place could boost learning and facilitate the encoding and future retrieval of the pieces of information. On the other hand, and considering that the participants in the current study were adult native speakers of the language, the developmental account of the context-dependent memory effect would predict minor, if not negative, object-context association effects derived from multiple pairings (e.g., different classroom settings) for the same element (e.g., a single word). Future studies should be aimed at elucidating whether these two opposing views could be responsible for the effects observed here, either testing children for which the context-dependent memory effect could be beneficial at their developmental stage or using a more fine-grained manipulation based on an incremental gradient of a number of contexts, as in Frances et al. [20]. Besides, it is worth considering the possibility that the type of perceptual manipulations carried out among the different environments in our experiment was insufficient to create episodic memory traces of the strength of those achieved in Tapia et al. [33]. This may have to do with how inherent perceptual variability is to the critical stimuli. In our experiment, perceptual variability was manipulated most heavily by means of changing the learning environment, that is, in the physical space in which participants found themselves. However, the type of manipulation used by Tapia et al. [33] had to do with the physical properties of the stimuli themselves (namely, the auditory carrier soundwaves the words were perceived through). One may say that perceptual variability of this kind is more likely to create episodic memory traces that are more tightly associated with the stimuli since attention cannot be focused away from them—while variability in the physical environment can arguably be ignored, in part, when the word display is in focus.

In this same line, and based on the information accumulation account of Ramscar et al. [52] and recent evidence from Johns et al. [53], it should be considered that age is also a factor that inherently increases experiential relationships with a progressively larger number of physical contexts. Older individuals are more familiar than younger ones with a wider range of contexts in which a word could be encountered. In this regard, individuals are predicted to become less attentive to the surrounding context as they gain expertise, given that learning involves disregarding unhelpful cues [54,55]. When selecting our VR environments, we ensured distinctiveness between each model; however, it should be noted that these were closely familiar to the type of physical environments participants spent most of their time in since primary education through university (namely, a series of classrooms) [33].

In a nutshell, in the current study, we were able to reproduce a highly immersive learning environment using VR, allowing for manipulations involving changes in the physical locations where learning took place. The present results confirmed no learning benefit associated with, or because of, the presentation of novel information across the different instances of the high environment diversity setting. As a result, the variety of the physical environments in which the presentation of the materials takes place does not appear to have a direct impact on the ability to learn through reading. However, when considering these results from the opposite perspective (namely, the lack of detrimental effects as a consequence of altering the learning environments), we could conclude that incidental learning appears to occur in the same manner regardless of the many learning scenarios in which the learning cues are offered. These findings pave the way for future applied learning studies in real-life environments (e.g., kindergartens, language learning facilities), where vocabulary acquisition and its evaluation can take place in several different locations.

## Figures and Tables

**Table 1:** Mean accuracy (proportion of correct responses) across conditions in the four experimental tasks. Standard deviations are provided in parentheses.

Evaluation Task	Stimulus Type	High Variability	Low Variability
Free recall		0.141(0.24)	0.125 (0.20)
Recognition	Old	0.741 (0.22)	0.714 (0.25)
	New	0.967 (0.09)	1 (0)
	New-related	0.716 (0.26)	0.678 (0.26)
Picture Matching	Match	0.707 (0.25)	0.768 (0.21)
	Competitor	0.774 (0.22)	0.719 (0.27)
	Distractor	0.580 (0.31)	0.580 (0.29)
	Unrelated	0.868 (0.22)	0.875 (0.23)
Sentence Completion		0.686 (0.22)	0.702 (0.20)

## Data Availability

All the data obtained in this study are available upon request to F.R. (jrocabado@nebrija.es).

## Supplementary Materials

The following supporting information can be downloaded at https://doi.org/10.6084/m9.figshare.21485619: Narrative texts used as materials in the incidental learning phase of the study.
